# Aluminum phosphate-modified red clay as a stable nanocomposite catalyst for the selective conversion of methanol to dimethyl ether

**DOI:** 10.1038/s41598-026-63442-x

**Published:** 2026-08-04

**Authors:** Mohamed N. Goda, Abd El-Aziz A. Said, Asmaa Mohamed, Esraa Magdy, Mohamed Abd El-Aal

**Affiliations:** 1 Chemistry Department, Faculty of Science, Assiut University, Assiut, 71516 Egypt; 2 Department of Chemistry, College of Science, Imam Mohammad Ibn Saud Islamic University (IMSIU), 11623 Riyadh, Saudi Arabia

**Keywords:** Aluminum phosphate, Red clay, Methanol, DME, Fuel, Chemistry, Engineering, Environmental sciences, Materials science

## Abstract

Aluminum phosphate-modified Egyptian red clay (AlPO_4_/ERC) nanocomposites were developed as low-cost solid acid catalysts for methanol dehydration to dimethyl ether (DME). Structural and physicochemical analyses (XRD, FTIR, N₂ adsorption–desorption, TEM, SEM, and acid-probe reactions) show that AlPO_4_ incorporation preserves the aluminosilicate framework while tuning surface acidity, porosity, and active-site accessibility. XRF and XRD confirm that ERC consists mainly of quartz, kaolinite, and iron oxides, whereas AlPO_4_ introduces additional phosphate phases that interact with the clay matrix. Catalytic testing reveals a strong dependence on AlPO_4_ loadings, with 5 wt.% AlPO_4_/ERC exhibiting optimal performance and achieving 90% methanol conversion with 100% DME selectivity at 200 °C. This enhancement correlates with an optimal balance of weak and intermediate acid sites (~ 2.5 mmol g^−1^) and a high specific surface area (52 m^2^ g^−1^), which maximizes the number of accessible active centers. Higher loadings reduce activity because of pore blockage and decreased site accessibility. Mechanistically, methanol dehydration proceeds via acid-catalyzed surface methoxy intermediates, with activity governed by acid-site distribution rather than iron species. The catalyst shows excellent stability over 15 days without deactivation, confirming its strong structural robustness. Overall, controlled AlPO_4_ incorporation optimizes the acidity-porosity synergy, enabling efficient and stable DME production.

**Supplementary Information:**

The online version contains supplementary material available at https://doi.org/10.1038/s41598-026-63442-x.

## Introduction

The development of renewable energy technologies as alternatives to fossil fuels, together with efforts to reduce harmful emissions, represents one of the most critical challenges in mitigating climate change. In this context, adopting sustainable and renewable energy sources has become essential for reducing environmental impacts. Abundant resources such as solar, wind, and biomass play a key role in supporting the transition toward a circular economy and lowering carbon emissions^1,2^.

Among the proposed alternative fuels, dimethyl ether (DME) has attracted significant attention as a clean substitute for conventional diesel fuel. DME is a colorless, non-toxic, non-corrosive, and environmentally benign compound with physical properties similar to those of liquefied petroleum gas (LPG)^3^. It also has a high cetane number (55–66), exceeding that of conventional diesel fuel and making it a promising fuel for compression-ignition engines^4^. In addition, DME combustion produces low NOx emissions, minimal smoke, and reduced engine noise, thereby enhancing its environmental and operational advantages. Apart from its use as a fuel, DME serves as a critical intermediate in the production of valuable chemicals, including lower olefins, methyl acetate, and dimethyl sulfate. It also functions as an aerosol propellant and a viable alternative to LPG^5^. Furthermore, DME can be synthesized from biomass-derived syngas, making it a renewable energy carrier.

One of the most efficient pathways for DME synthesis is the dehydration of methanol. This process has been extensively studied using a wide variety of solid acid nanocomposites, including γ-Al_2_O_3_, zeolites, silica-modified alumina, mixed metal oxides, TiO_2_–ZrO_2_, and mesoporous silicates^6–8^. Despite their high catalytic activity, these materials suffer from several limitations. The highly exothermic nature of methanol dehydration can generate hot spots within the catalyst bed, leading to catalyst deactivation. Furthermore, carbonaceous deposits may accumulate on the catalyst surface, blocking active sites, while the water produced during the reaction competes with methanol for acidic sites, resulting in a decline in catalytic performance. Consequently, the development of new nanocomposites with improved stability and resistance to deactivation remains an active area of research.

Natural clay minerals, composed mainly of phyllosilicates, have emerged as promising catalytic materials because of their abundance, low cost, and environmental friendliness^9^. Clay-based nanocomposites have been widely explored for applications such as methanol-to-DME conversion, hydrogen production, photocatalysis, and environmental remediation^10–13^. Their advantageous properties include a high surface area, porosity, cation-exchange capacity, and excellent thermal and chemical stability^14,15^. Moreover, their structures can be modified through various treatments to enhance porosity and increase the number of active sites, thereby improving catalytic performance^16,17^.

Aluminum phosphate (AlPO_4_) is another important catalytic material with tunable acid–base properties, making it suitable for a wide range of reactions, including dehydration, isomerization, and alkylation^18,19^. Owing to its structural similarity to silica, AlPO_4_ has also been widely investigated as a catalyst and catalyst support in various catalytic systems^18,19^.

In our previous studies, Egyptian red clay (ERC) demonstrated promising catalytic performance. For instance, ERC calcined at 300 °C showed excellent activity in the gas-phase synthesis of bio-ethyl acetate, achieving 98% conversion with complete selectivity^20^. This performance was attributed to the presence of medium-strength Brønsted acid sites. Additionally, ERC-supported γ-Al_2_O_3_ nanocomposites exhibited high efficiency in methanol dehydration, with 5% HCl/10% γ-Al_2_O_3_/ERC achieving 90% conversion and complete selectivity to DME at 200 °C^12^. More recently, ZrO_2_-modified ERC nanocomposites also showed outstanding activity, reaching 94% methanol conversion with full selectivity at 250 °C^21^. These findings highlight the crucial role of surface acidity in determining catalytic performance.

Despite these advances, no studies have reported the use of AlPO_4_ supported on ERC for the dehydration of methanol to DME. Therefore, the present work aims to develop and evaluate AlPO_4_/ERC as an efficient solid acid nanocomposite for highly selective DME production with high selectivity at relatively low temperatures. The prepared nanocomposites were comprehensively characterized using XRD, SEM, FTIR, TEM, N₂ adsorption–desorption, and acidity measurements. Furthermore, the influence of operating conditions on catalytic performance was systematically investigated, with particular emphasis on the relationship between catalytic activity and the nature of the surface acid sites.

## Materials and methods

### Materials

Natural Egyptian red clay (ERC) was obtained from the El-Kharga region, New Valley, Western Desert, Egypt. The raw clay was dried and sieved to 0.125 mm using a stainless-steel mesh (ANALYSENSIEB, Germany) before use. All reagents were of analytical grade and used without further purification. These included aluminum nitrate nonahydrate (Al(NO₃)₃·9H₂O, 98%, Alpha Chemika), diammonium hydrogen phosphate ((NH₄)₂HPO₄, 98%, Alpha Chemika), methanol (CH₃OH, 99.9%, Alfa Chemical), and isopropyl alcohol (99.9%, Cambrian Chemicals). Pyridine (99%, Sigma-Aldrich) and 2,6-dimethylpyridine (99%, Sigma-Aldrich) were also used for catalyst acidity characterization.

### Synthesis of AlPO_4_/ERC nanocomposites

**Scheme 1:**
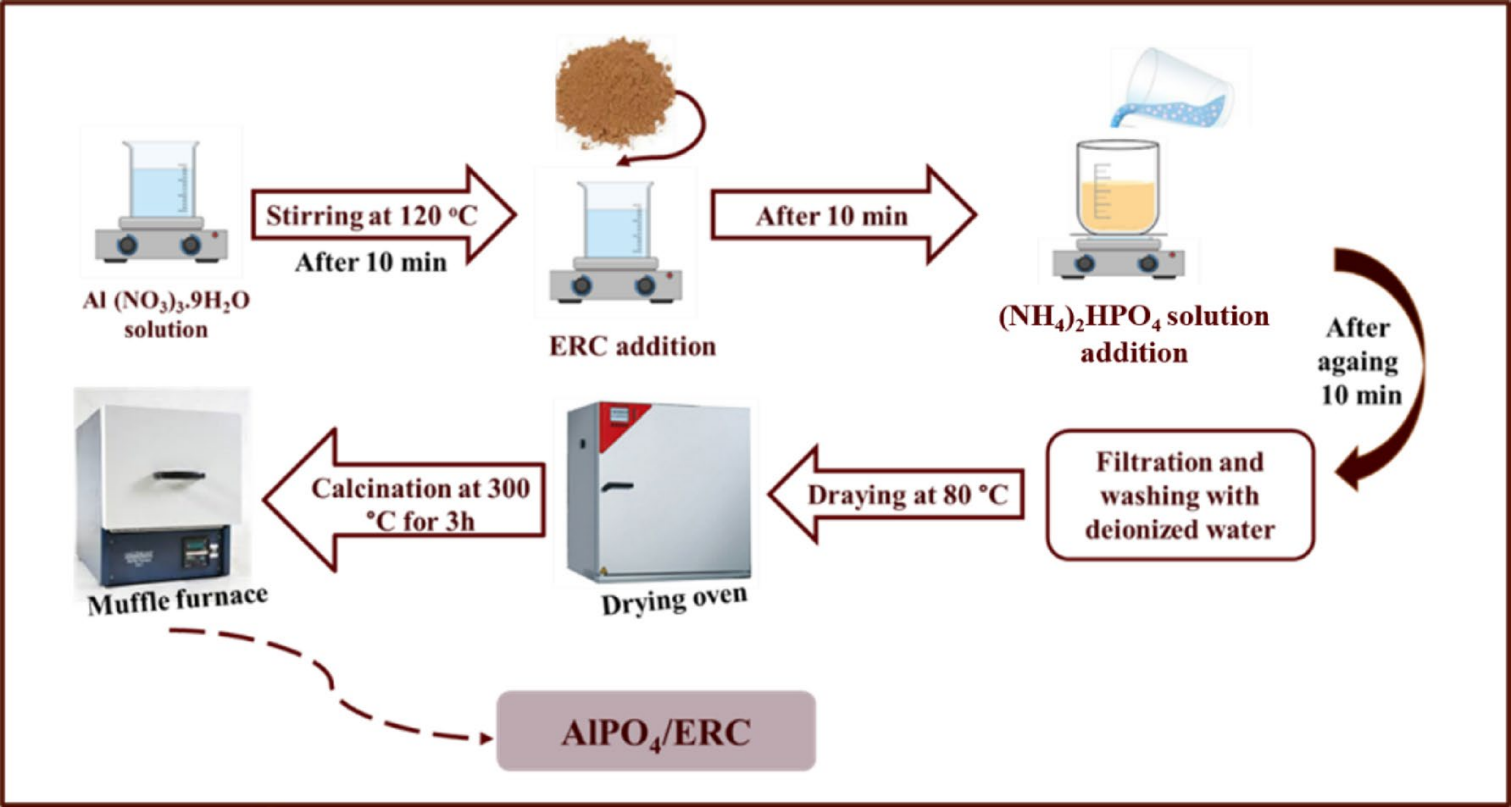
Flow chart for the synthesis of AlPO_4_/ERC nanocomposites.

AlPO_4_/ERC nanocomposites with varying AlPO_4_ contents (3–30 wt.%), were synthesized via a precipitation method^22^, as shown in Scheme 1. For the 5% AlPO_4_/ERC nanocomposite, 4.6 g of Al(NO₃)₃·9H₂O was dissolved in 100 mL of deionized water and heated at 120 °C with stirring for 10 min. Subsequently, 28.5 g of ERC was added under continuous stirring, followed by the dropwise addition of an aqueous (NH₄)₂HPO₄ solution (1.62 g in 50 mL of deionized water). The suspension was aged for 10 min and then cooled to room temperature. The precipitate was filtered, washed thoroughly with deionized water, dried at 80 °C overnight, and calcined at 300 °C for 3 h in static air. The untreated ERC sample was also calcined at 300 °C before characterization to enable a direct and consistent comparison with the AlPO_4_-modified nanocomposites. The most active nanocomposite catalyst was further calcined at 500 and 600 °C to investigate the influence of calcination temperature on its physicochemical properties and catalytic performance.

### Characterization techniques

#### Nanocomposite characterization

The chemical composition of the ERC was analyzed by X-ray fluorescence (XRF) at CEMEX, Assiut. The phase structure of the nanocomposites was analyzed by X-ray diffraction (XRD) using a Philips PW 2103/00 diffractometer with Cu Kα radiation (λ = 1.5418 Å) and a Ni filter, scanning over a 2θ range of 4–80°, with a step size of ~ 0.02° and an appropriate scan rate to ensure a good signal-to-noise ratio. The crystallite size (*D*) was calculated using the Scherrer equation^23–25^: $D=k\lambda /\beta cos\theta$, where *D* represents the average crystallite size (nm), K is the shape factor, which is commonly taken as 0.9, λ is the X-ray wavelength (1.5406 Å for Cu Kα radiation), β is the full width at half maximum (FWHM) of the selected diffraction peak expressed in radians, and θ is the Bragg angle, defined as half of the measured 2θ value. Fourier transform infrared (FTIR) spectra were recorded using a Nicolet 6700 spectrophotometer over the range of 4000–400 cm^−1^. Nitrogen adsorption–desorption isotherms were measured at − 196 °C using a Quantachrome NOVA 3200 analyzer to determine the specific surface area (S_BET_) and pore characteristics of the ERC and the AlPO_4_/ERC nanocomposites^26^. The surface area was calculated using the Brunauer–Emmett–Teller (BET) method over the relative-pressure (P/P^₀^) range of 0.05–0.30, whereas the pore volume and pore-size distribution were obtained using the Barrett–Joyner–Halenda (BJH) model. The BJH pore size distribution analysis was determined using the adsorption branch of the nitrogen adsorption–desorption isotherm. The morphology of the synthesized catalysts was examined using a scanning electron microscope (SEM; TESCAN VEGA 3 SB) equipped with an energy-dispersive X-ray spectroscopy (EDX) detector for elemental composition analysis. The morphology and particle size of the samples were examined by transmission electron microscopy (TEM) using a JEOL JSM-5400LV instrument (JEOL, Tokyo, Japan).

### Acidity determination

To evaluate catalyst acidity, isopropyl alcohol (IPA) dehydration experiments and adsorption experiments with pyridine (PY) and 2,6-dimethylpyridine (DMPY) were conducted. The IPA dehydration test was performed in a conventional fixed-bed Pyrex flow reactor at atmospheric pressure using nitrogen as the carrier gas. In a typical run, 500 mg of catalyst was loaded, and a feed containing 2% IPA was passed over the catalyst at a total flow rate of 50 mL min^−1^ and a reaction temperature of 150 °C. The propene yield (%) was recorded after 1 h on stream, once steady-state conditions had been reached. The effluent gases were analyzed directly using a Unicam ProGC gas chromatograph equipped with a flame ionization detector (FID) and a 2 m column containing 10% PEG 400. The nature and strength of the acid sites were further probed using PY and DMPY chemisorption according to previously reported procedures^27^.

The acid-site densities of the ERC and the modified ERC were determined using an acid–base titration method^28^. The samples were first dried at 110 °C for 4 h to remove any physically adsorbed moisture and then cooled in a desiccator before analysis. Approximately 0.3 g of each sample with a different AlPO_4_ loading (3–30 wt.%) was accurately weighed and transferred into a clean Erlenmeyer flask. A known excess volume (25.00 mL) of standardized 0.05 M NaOH solution was then added to each flask containing the catalyst sample. The flasks were sealed and continuously stirred for 12 h at room temperature to ensure complete neutralization of the acidic sites by NaOH. After equilibration, the solid catalyst was separated from the liquid phase by filtration, and the clear filtrate was collected. Subsequently, 2–3 drops of phenolphthalein indicator were added to the filtrate, and the remaining unreacted NaOH was titrated with standardized 0.05 M HCl solution until the pale pink color was obtained. A blank experiment was performed under identical conditions without the addition of a catalyst, using the same volume and concentration of NaOH solution. The amount of acid sites was calculated from the difference between the volumes of HCl consumed in the blank and sample titrations according to Eq. (1):

1 $${\boldsymbol{n}}_{{{\boldsymbol{acid}}}} = \left( {{\boldsymbol{V}}_{{\boldsymbol{b}}} - {\boldsymbol{V}}_{{\boldsymbol{s}}} } \right) \times {\boldsymbol{C}}$$

where V_b_ and V_s_ represent the volumes of HCl used for the blank and sample, respectively, and (C) is the concentration of HCl.

The acid site density of the catalyst was then determined using Eq. (2):

2 $${\text{Acid site density}} = \frac{{{\boldsymbol{n}}_{{{\boldsymbol{acid}}}} }}{{\boldsymbol{m}}}{ }$$

where m is the mass of the catalyst sample.

### Catalytic performance evaluation

The catalytic performance for DME production was evaluated in a conventional fixed-bed flow reactor using 500 mg of either pure ERC or an AlPO_4_-modified ERC catalyst. The reaction was conducted at atmospheric pressure with a gas hourly space velocity (GHSV) of 6000 h^−1^ and a feed containing 4% methanol over a temperature range of 200–350 °C. The products were analyzed using a Unicam ProGC gas chromatograph equipped with a flame ionization detector (FID) and a 2 m DNP glass column. For each experiment, at least three consecutive measurements were recorded under steady-state conditions after 1 h on stream following methanol introduction. The averaged values were used to determine methanol conversion (%) and DME selectivity (%). Methanol conversion and DME selectivity were calculated using the following equations^29^.

3 $${\mathbf{Methanol}} {\mathbf{Conversion}} \left( \% \right) = \frac{{\left[ {{\mathbf{Methanol}}} \right]_{{{\mathbf{in}}}} - \left[ {{\mathbf{Methanol}}} \right]_{{{\mathbf{out}}}} }}{{\left[ {{\mathbf{Methanol}}} \right]_{{{\mathbf{in}}}} }} {\mathbf{x}} 100$$

4 $${\mathbf{Selectivity}} {\mathbf{of}} {\mathbf{DME}} \left( \% \right) = \frac{{\left[ {{\mathbf{DME}}} \right]}}{{\left[ {{\mathbf{DME}}} \right] + \left[ {{\mathbf{Other}} {\mathbf{Products}}} \right]}} {\mathbf{x}} 100$$

5 $${\mathbf{Yield}} {\mathbf{of}} {\mathbf{DME}} \left( \% \right) = \frac{{{\mathbf{Methanol}} {\mathbf{Conversion}} \times {\mathbf{Selectivity}} {\mathbf{of}} {\mathbf{DME}}}}{100}$$

## Results and discussion

### Characterization of the nanocomposite

The XRF composition of the raw ERC material is summarized in Table S1^20^ as bulk oxide weight percentages (wt.%). The major constituents of ERC are silica, alumina, and iron oxides, indicating a high clay-mineral content in the investigated sample. In contrast, oxides such as Na_2_O, MnO_2_, MgO, K₂O, CaO, and TiO_2_ are present at relatively low concentrations. Furthermore, the SiO_2_/Al_2_O_3_ ratio was calculated to be approximately 1.11, which is greater than 1.0.

**Fig. 1:**
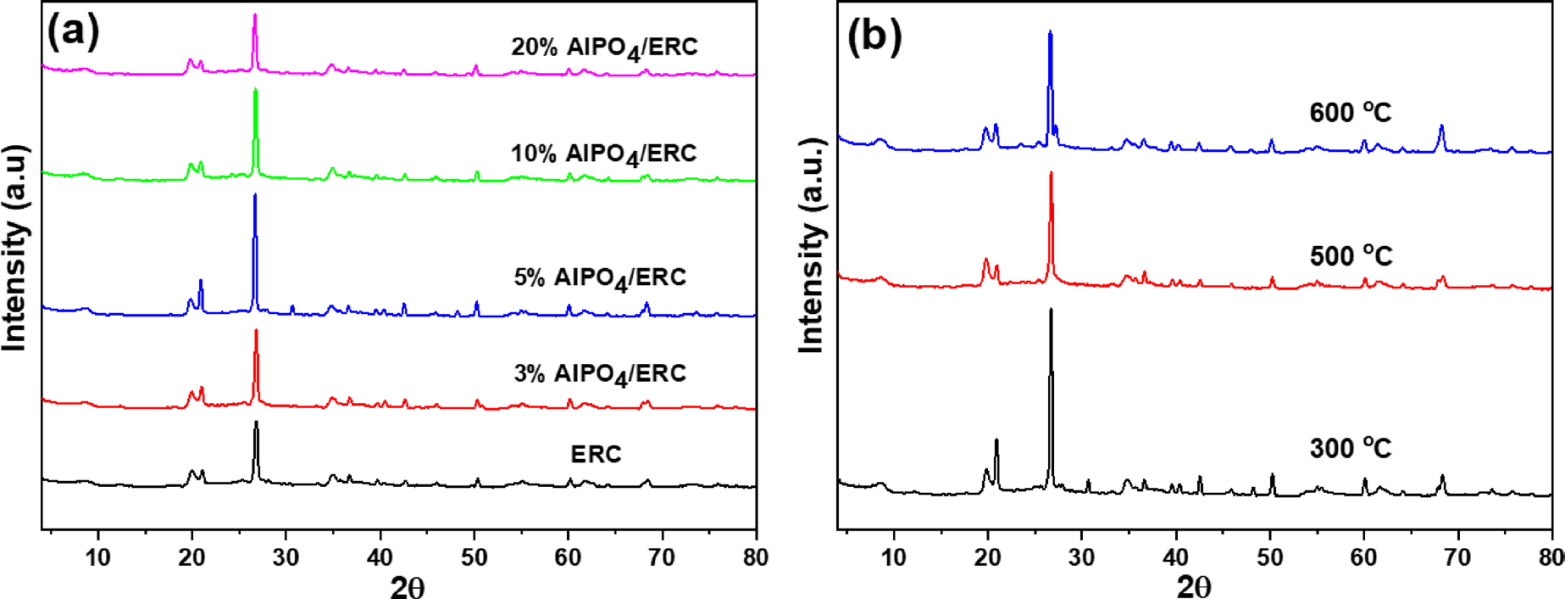
XRD patterns of **(a)** pure ERC adapted from Refs.^12,21^, and 3–20 wt.% AlPO_4_/ERC nanocomposites and **(b)** 5% AlPO_4_/ERC nanocomposites treated at different calcination temperatures.

The X-ray diffraction (XRD) patterns of pristine ERC and AlPO_4_/ERC nanocomposites (3–20 wt.%) calcined at 300 °C are presented in Fig. 1a. The diffraction pattern of untreated ERC (as previously published in^12,21^) shows characteristic reflections at 2θ = 21, 26.8, 36.7, 50.3, 60.2, and 68.4°, which are assigned to hexagonal quartz (space group P3₁21, JCPDS No. 00–003-0444). Additional reflections at 19.9, 25.3, 34.9, 39.7, 55, and 61.7° correspond to kaolinite^20^, whereas the peak at 33.3° is attributed to rhombohedral hematite (Fe_2_O_3_, space group R-3c, JCPDS No. 00–001-1053). Minor phases such as siderite and goethite are also detected at 42.7 and 64.2°, respectively^12^. The XRD pattern of pure AlPO_4_ exhibits distinct diffraction peaks at 2θ = 20.1, 21.3, 23.1, 25.4, 35.4, 53.1, and 72.2°, which are characteristic of crystalline AlPO_4_^30^. Notably, the berlinite structure of AlPO_4_ is isostructural with quartz^20,29,31^, which explains the overlap of their diffraction features. At low AlPO_4_ loadings (3 and 5 wt.%), the XRD profiles remain largely similar to those of pristine ERC, with slight peak shifts and increased intensities, particularly around 20.9° and 26.6°. This enhancement is likely due to the superposition of diffraction peaks from the quartz and AlPO_4_ phases^32^. As the AlPO_4_ content increases (≥ 10 wt.%), a gradual decrease in peak intensity is observed, which may be attributed to partial coverage or dilution of the quartz phase^33^. Concurrently, a reduction in kaolinite crystallinity is evident, suggesting structural perturbation of the clay matrix upon phosphate incorporation^34^. Figure 1b shows the XRD patterns of the 5% AlPO_4_/ERC nanocomposite calcined at different temperatures. Increasing the calcination temperature to 500 and 600 °C results in a slight decrease in peak intensity compared with that of the sample treated at 300 °C, which may be associated with the formation of amorphous silica-phosphate species or phase aggregation^35^. The average crystallite sizes (*D*) of ERC and the AlPO_4_-modified nanocomposites calcined at 300 °C were calculated to be 21.4, 24.8, 20.6, 19.3, and 21.0 nm for pristine ERC and the 3%, 5%, 10%, and 20% AlPO_4_/ERC nanocomposites, respectively. For the 5% AlPO_4_/ERC nanocomposite calcined at higher temperatures, the crystallite sizes increased slightly to 22.6 nm and 22.3 nm at 500 and 600 °C, respectively.

### FTIR analysis

**Fig. 2:**
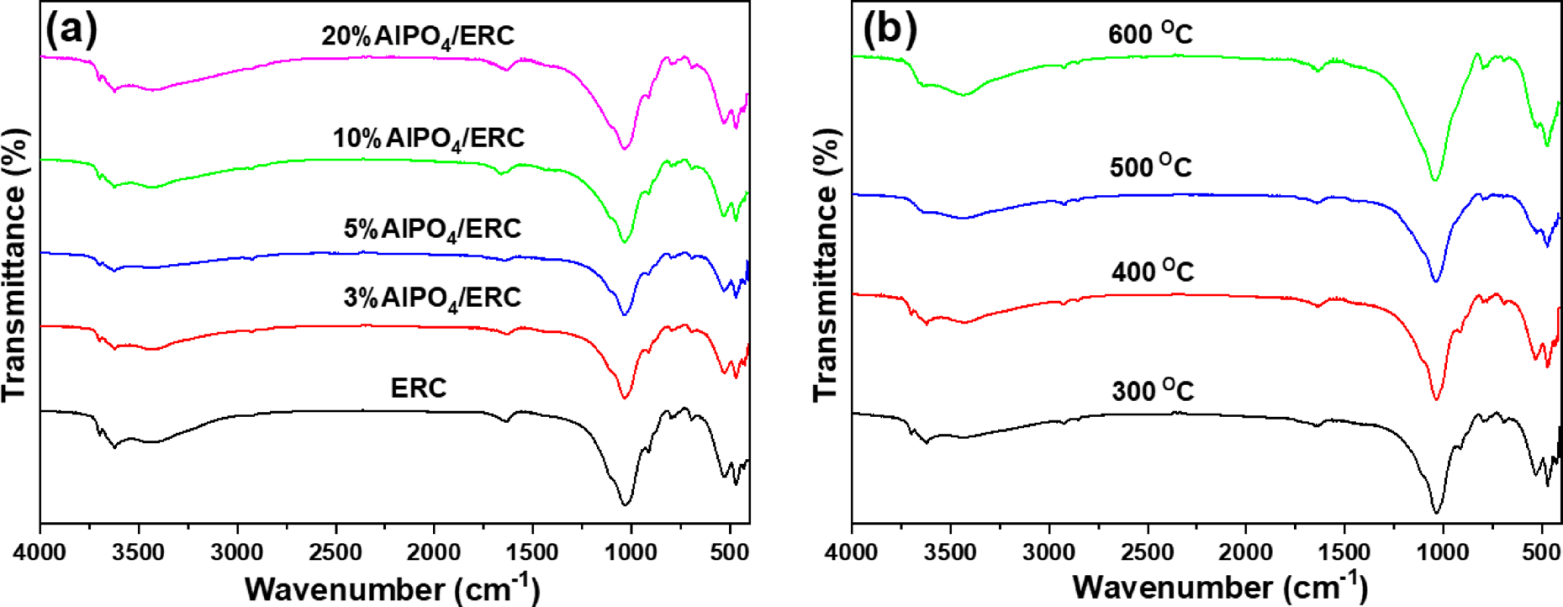
FTIR spectra of **(a)** untreated ERC (adapted from Refs ^12,21^.) and AlPO_4_/ERC nanocomposites with loadings of 3–20 wt.% calcined at 300 °C, and **(b)** the 5% AlPO_4_/ERC nanocomposite calcined at various temperatures.

The FTIR spectra of pristine ERC and AlPO_4_/ERC nanocomposites (3–20 wt.%) calcined at 300 °C are presented in Fig. 2a. The spectrum of untreated ERC exhibits four well-defined O–H stretching bands. The bands at 3698 and 3621 cm^−1^ are attributed to Si–OH and Al–OH groups, characteristic of the kaolinite structure. The presence of adsorbed water is evidenced by bands at 3440 cm^−1^ (O–H stretching) and 1640 cm⁻^1^ (H–O–H bending)^36,37^. Weak bands observed at 2923 and 2852 cm^−1^ are assigned to the C–H stretching vibrations of residual organic species^38^. The strong band at 1031 cm^−1^ corresponds to Si–O stretching vibrations, whereas the band at 912 cm^−1^ is associated with Al–OH bending modes^37,39^. Additional bands at 798 cm^−1^ (Si–O) and 694 cm^−1^ (Si–O–Al) are related to framework vibrations^40^, whereas those at 521 cm⁻^1^ (Al–O–Si) and 467 cm^−1^ (Si–O–Si) arise from bending modes of the aluminosilicate structure^37,40^. The FTIR spectra of the AlPO_4_/ERC nanocomposites with varying loadings (3–20 wt.%) exhibit features similar to those of pristine ERC, indicating that the fundamental clay structure is largely preserved after modification. The characteristic phosphate vibrations of AlPO_4_ are not distinctly resolved, likely because they overlap with the intrinsic silicate and aluminate bands of the clay matrix. The influence of calcination temperature on the 5% AlPO_4_/ERC nanocomposite is shown in Fig. 2b. The intensity of the band at 1031 cm^−1^ progressively increases with increasing calcination temperature is observed, suggesting structural reorganization and a possible enhancement in Si–O network ordering.

**Fig. 3:**
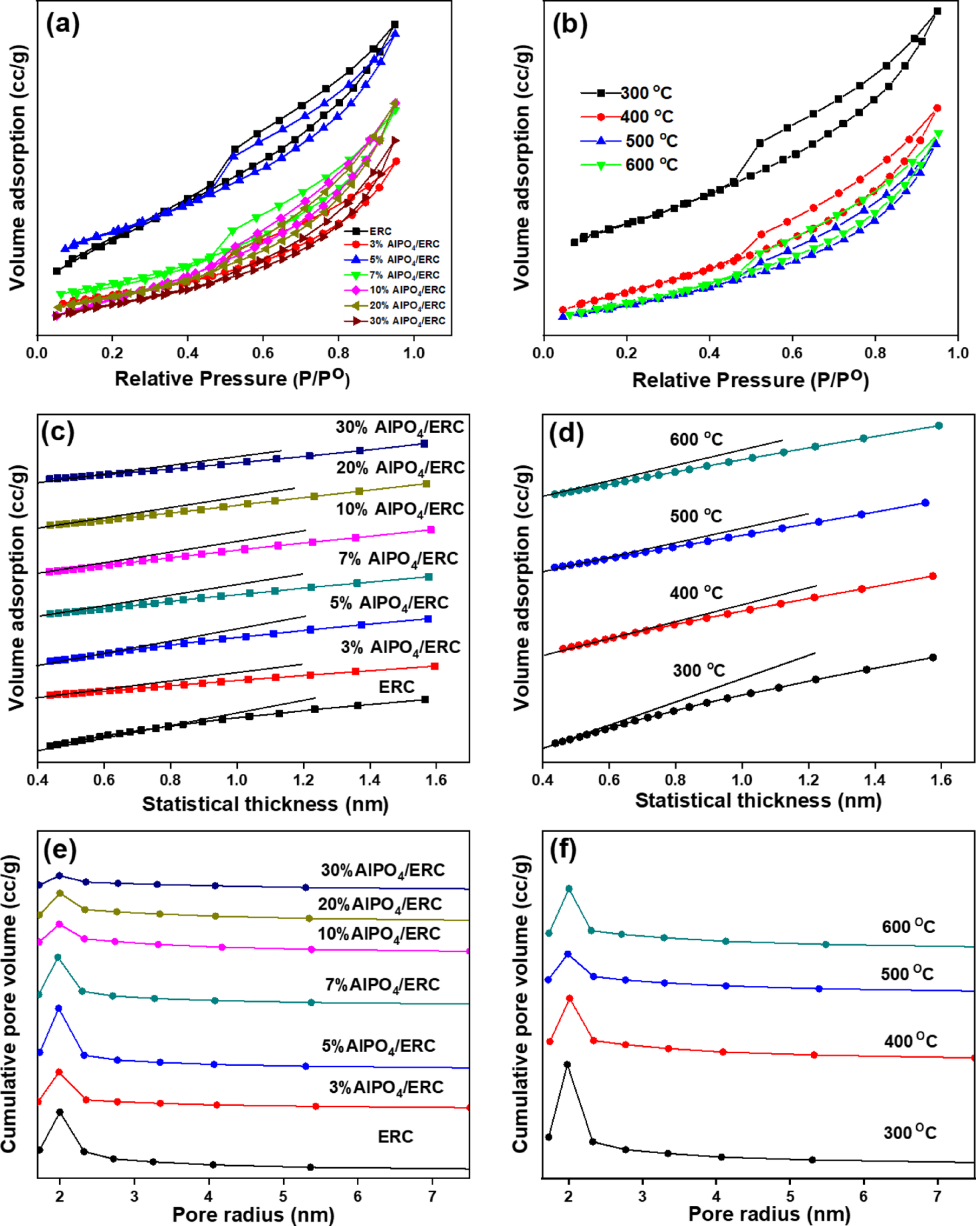
N₂ adsorption–desorption isotherms and related textural properties: **(a)** isotherms of untreated ERC adapted from Refs^12,21^. and 3–30% AlPO_4_/ERC nanocomposites calcined at 300 °C; **(b)** isotherms of 5% AlPO_4_/ERC nanocomposites calcined at different calcination temperatures; **(c)** V_a–t_ plots of untreated ERC and 3–30% AlPO_4_/ERC, **(d)** V_a–t_ plots of 5% AlPO_4_/ERC calcined at different calcination temperatures; **(e)** PSD of pure ERC and 3–30% AlPO_4_/ERC; and **(f)** PSDs of 5% AlPO_4_/ERC calcined at different calcination temperatures.

The textural characteristics of the nanocomposites, including specific surface area and porosity, were assessed using nitrogen adsorption–desorption measurements^44^. Figure 3a shows the N₂ adsorption–desorption isotherms of untreated ERC and 3–30% AlPO_4_/ERC nanocomposites that were calcined at 300 °C. All samples exhibit type II isotherms with H3 hysteresis loops, indicating the presence of non-rigid aggregates of plate-like particles, according to the IUPAC and de Boer classifications^41^. This hysteresis behavior is characteristic of mesoporous structures with slit-shaped pores and may also indicate the presence of minor microporosity^27^. The N₂ adsorption–desorption isotherms of the 5% AlPO_4_/ERC nanocomposite calcined at various temperatures (300–600 °C) are shown in Fig. 3b. The persistence of type II isotherms with H3 hysteresis loops at all calcination temperatures suggests that the overall porous structure of the nanocomposite remains largely stable during thermal treatment. Further insight into the porous structure was obtained from V_a-t_-plots and pore-size distribution (PSD) analyses. The V_a-t_-plots (Fig. 3c, d) exhibit downward deviations from linearity, confirming the coexistence of micro- and mesopores within the nanocomposite structure. The BJH pore-size distributions of pristine ERC and the AlPO_4_/ERC nanocomposites (3–30 wt.%) calcined at 300 °C (Fig. 3e) reveal a unimodal distribution centered at ~ 2 nm, confirming their predominantly mesoporous nature. Similarly, the PSD of the 5% AlPO4/ERC nanocomposite remains virtually unchanged over the calcination-temperature range of 300–600 °C (Fig. 3f), suggesting that thermal treatment does not significantly affect the pore structure^12^.

The textural properties of pristine ERC and the AlPO_4_/ERC nanocomposites (3–30 wt.%) calcined at 300 °C, together with those of the 5% AlPO_4_/ERC samples calcined at different temperatures, are presented in Table 1. Untreated ERC exhibits a specific surface area (S_BET_) of 53.1 m^2^ g^−1^, in agreement with previous reports^12,21^. The S_BET_ decreases to 25.2 m^2^ g^−1^ after the addition of 3 wt.% AlPO_4_, with an average pore width of 1.98 nm and a total pore volume of 0.029 cm^3^ g⁻^1^. This reduction can be attributed to partial coverage of the ERC surface by phosphate species, which leads to pore blockage and a decrease in the accessible surface area^35^. At an AlPO_4_ loading of 5 wt.%, both S_BET_ and pore volume increase significantly to 52.0 m^2^ g^−1^ and 0.044 cm^3^ g^−1^, respectively. This improvement may be attributed to the creation of new porous structures through interactions between aluminum and phosphate species, which promote pore development and surface reorganization^35^. However, a further increase in AlPO_4_ content beyond 5 wt.% results in a progressive decline in S_BET_, reaching 22.9 m^2^ g^−1^ at 30 wt.%, together with a reduction in pore volume to 0.032 cm^3^ g^−1^. This behavior is likely due to excessive phosphate accumulation, which blocks pore channels and occupies surface sites, thereby reducing accessible porosity^19^. The effect of calcination temperature on the 5% AlPO_4_/ERC nanocomposite was also examined. S_BET_ decreased significantly from 52.0 to 22.7 m^2^ g^−1^ as the temperature increased from 300 to 600 °C. This decrease can be explained by sintering processes and partial pore collapse at high temperatures. Additional textural parameters obtained from the t-plot analysis are presented in Fig. 3 and Table 1. The close agreement between the external surface area (S_t_) and S_BET_ values confirms the reliability of the selected standard t-curve.

**Table 1:** Textural properties of untreated ERC and AlPO_4_/ERC nanocomposites with different AlPO_4_ loadings (3–30 wt.%) calcined at 300 °C, and of 5 wt.% AlPO_4_/ERC calcined at various temperatures.

Nanocomposites	S_BET_ (m^2^ g^−1^)	S_t_ (m^2^ g^−1^)	Total pore volume (cc g^−1^)	Average pore diameter (nm)	Ρ (g/cm^3^)	*D*_BET_ (nm)
Untreated ERC estimated from^12,21^	53.1	53.1	0.046	1.99	2.600	43.4
3% AlPO_4_/ERC	25.2	25.2	0.029	1.99	2.597	91.6
5% AlPO_4_/ERC	52.01	52.01	0.044	1.98	2.595	44.4
7% AlPO_4_/ERC	30.66	30.66	0.038	1.97	2.592	74.4
10% AlPO_4_/ERC	26.88	26.88	0.040	1.99	2.588	86.2
20% AlPO_4_/ERC	25.64	25.64	0.041	1.99	2.576	90.8
30% AlPO_4_/ERC	22.91	22.91	0.032	1.99	2.563	102
5%AlPO_4_/ERC (400 °C)	27.59	27.59	0.036	2.01	2.596	83.8
5%AlPO_4_/ERC (500 °C)	21.88	21.88	0.031	1.99	2.598	105.5
5%AlPO_4_/ERC (600 °C)	22.69	22.69	0.033	2.00	2.600	102

Equation 6 was used to estimate the theoretical particle sizes of the nanocomposites, which are also listed in Table 1. As the AlPO_4_ loading has increased to 30 wt.%, the estimated particle size increased markedly from 43.4 nm for pristine ERC to 102 nm for the 30 wt.% AlPO4/ERC nanocomposite. Similarly, increasing the calcination temperature of the 5% AlPO_4_/ERC nanocomposite from 300 to 600 °C increased the particle size from 44.4 to 102 nm, consistent with thermal sintering and particle growth at higher temperatures.

6 $$D = \frac{6000}{{\rho * S_{BET} }}$$

**Fig. 4:**
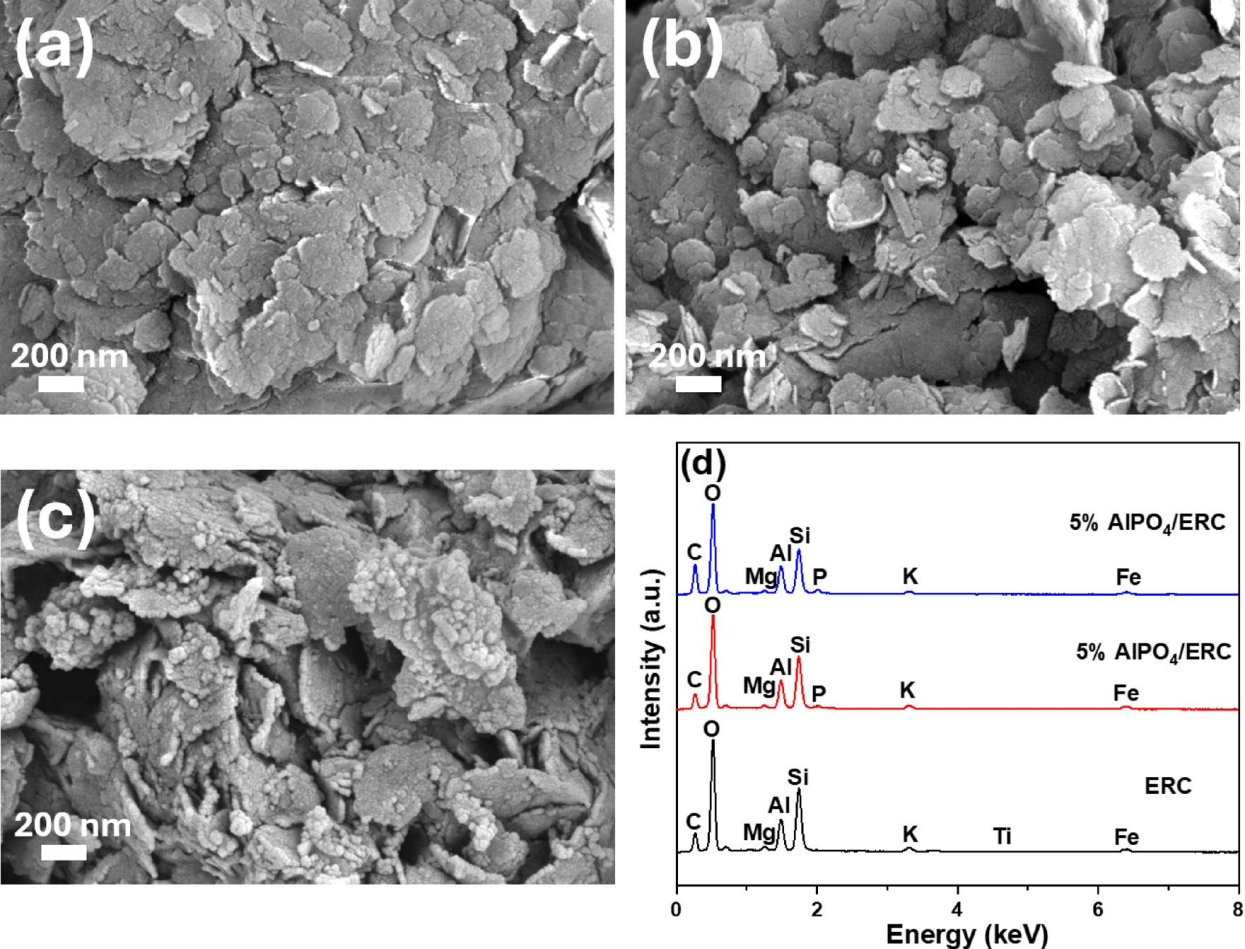
SEM images of **(a)** ERC, **(b)** 5% AlPO_4_/ERC, and **(c)** 10% AlPO_4_/ERC nanocomposites, and **(d)** EDX spectra of ERC and the 5% and 10% AlPO_4_/ERC nanocomposites.

The SEM micrographs and EDX analyses of pure ERC and the 5% and 10% AlPO_4_/ERC nanocomposites calcined at 300 ºC are presented in Fig. 4. The SEM image of pure ERC (Fig. 4a) reveals a compact, flaky, and layered morphology composed of irregular plate-like particles stacked on one another, which is characteristic of naturally occurring clay minerals. The surface appears relatively smooth, with dense agglomeration of the flakes and limited visible porosity. After loading with 5% AlPO_4_ (Fig. 4b), the general flaky structure of the clay remains largely preserved, indicating that the incorporation of a small amount of AlPO_4_ does not significantly disturb the structural framework of the clay support. However, the surface becomes slightly rougher and less compact, accompanied by the appearance of small, dispersed particles and partial exfoliation of the clay layers, suggesting the initial deposition of AlPO_4_ species on the ERC surface. Furthermore, some interparticle voids and pores become more visible than in pure ERC, which may improve the accessibility of the active sites. A significant morphological change is observed after increasing the AlPO_4_ loading to 10% (Fig. 4c). The clay surface becomes heavily decorated with numerous fine spherical and granular particles distributed over the flaky sheets. These particles are attributed to deposited AlPO_4_ species and indicate successful loading and greater surface coverage of AlPO_4_ on the clay matrix. In addition, the clay layers appeared more separated and porous, with the formation of interconnected voids and a rough, heterogeneous texture.

The EDX spectra of pure ERC and the 5% and 10% AlPO_4_/ERC nanocomposites calcined at 300 ºC are shown in Fig. 4d. The EDX analysis provides information on the elemental composition and confirms the successful incorporation of AlPO_4_ into the ERC structure. The spectrum of pure ERC exhibits characteristic peaks corresponding to carbon (C), oxygen (O), magnesium (Mg), aluminum (Al), silicon (Si), potassium (K), titanium (Ti), and iron (Fe), which are commonly detected in natural clay minerals. The same major elements were detected in the AlPO_4_/ERC nanocomposites, although the Ti peak disappears after AlPO_4_ loading, possibly because of its low concentration and partial surface coverage by AlPO_4_ particles. Importantly, a new peak corresponding to phosphorus (P) appears in the spectra of both the 5% and 10% AlPO_4_/ERC nanocomposites, providing strong evidence of the successful incorporation of AlPO_4_ onto the ERC surface. Moreover, the intensity of the P peak increases noticeably with increasing AlPO_4_ loading percentage, indicating a progressive increase in the amount of deposited phosphate species. A slight increase in the Al content is also observed after loading, which further supports the formation of AlPO_4_ on the clay surface. The quantitative elemental compositions obtained from the EDX analysis are summarized in Table 2. The results indicate that all catalysts contain high proportions of C, O, Si, Al, and Fe, whereas Mg, K, and Ti are present in relatively minor amounts. Pure ERC shows no detectable phosphorus content, whereas both the Al and P percentages increased with increasing AlPO_4_ loading. Simultaneously, slight decreases in some native clay elements, particularly Si, Fe, and Ti, are observed because of the partial surface coverage of the ERC surface by AlPO_4_ species. Collectively, these findings confirm the successful loading and dispersion of AlPO_4_ onto the ERC surface, particularly at higher loading percentages.

**Table 2:** Chemical composition of ERC, the 5% and 10% AlPO_4_/ERC nanocomposites calcined at 300 °C.

Element	Catalyst					
	ERC		5% AlPO_4_/ERC		10% AlPO_4_/ERC	
	Weight (%)	Atomic (%)	Weight (%)	Atomic (%)	Weight (%)	Atomic (%)
C	30.7	40.9	31.6	41.35	39.5	50.5
O	48.4	48.4	47.6	47.9	43.4	41.1
Mg	0.6	0.4	0.4	0.3	0.3	0.2
Al	4.9	2.9	5.1	3.0	5.3	3.1
Si	10.0	5.7	9.9	5.6	6.8	3.6
K	1.3	0.5	1.3	0.5	0.9	0.4
Ti	0.2	0.1	0.2	0.1	0.0	0.0
Fe	3.8	1.1	3.4	0.95	3.0	0.8
P	0.0	0.0	0.5	0.3	0.7	0.3

**Fig. 5:**
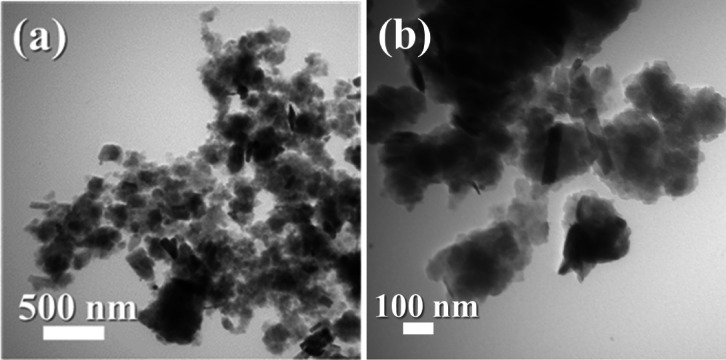
TEM images of **(a)** untreated ERC adapted from Refs ^12,21^.) and **(b)** 5% AlPO_4_/ERC calcined at 300 °C.

TEM was used to examine the surface morphology and microstructural characteristics of pristine ERC and the 5% AlPO_4_/ERC nanocomposites calcined at 300 °C^42^, as illustrated in Fig. 5. Untreated ERC exhibits dense particles and irregularly shaped agglomerates with sizes ranging from 180 to 354 nm, consistent with earlier studies^12,21^ (Fig. 5a). The presence of these large, compact particles is likely due to aluminosilicate frameworks that are partially coated or associated with finely dispersed iron oxide phases^43^. For the 5% AlPO_4_/ERC nanocomposite (Fig. 5b), the TEM image reveals more fragmented, less compact aggregates, with an average particle size of approximately 213 nm. This reduction indicates that the incorporation of AlPO_4_ leads to partial disaggregation and improved dispersion of the ERC aggregates. The observed morphological changes are consistent with the XRD and textural-analysis results, confirming that AlPO_4_ modification influences the structural organization of the clay matrix. Overall, the TEM analysis reveals a clear trend toward reduced particle size and improved dispersion following AlPO_4_ incorporation.

### Acidity determination

**Fig. 6:**
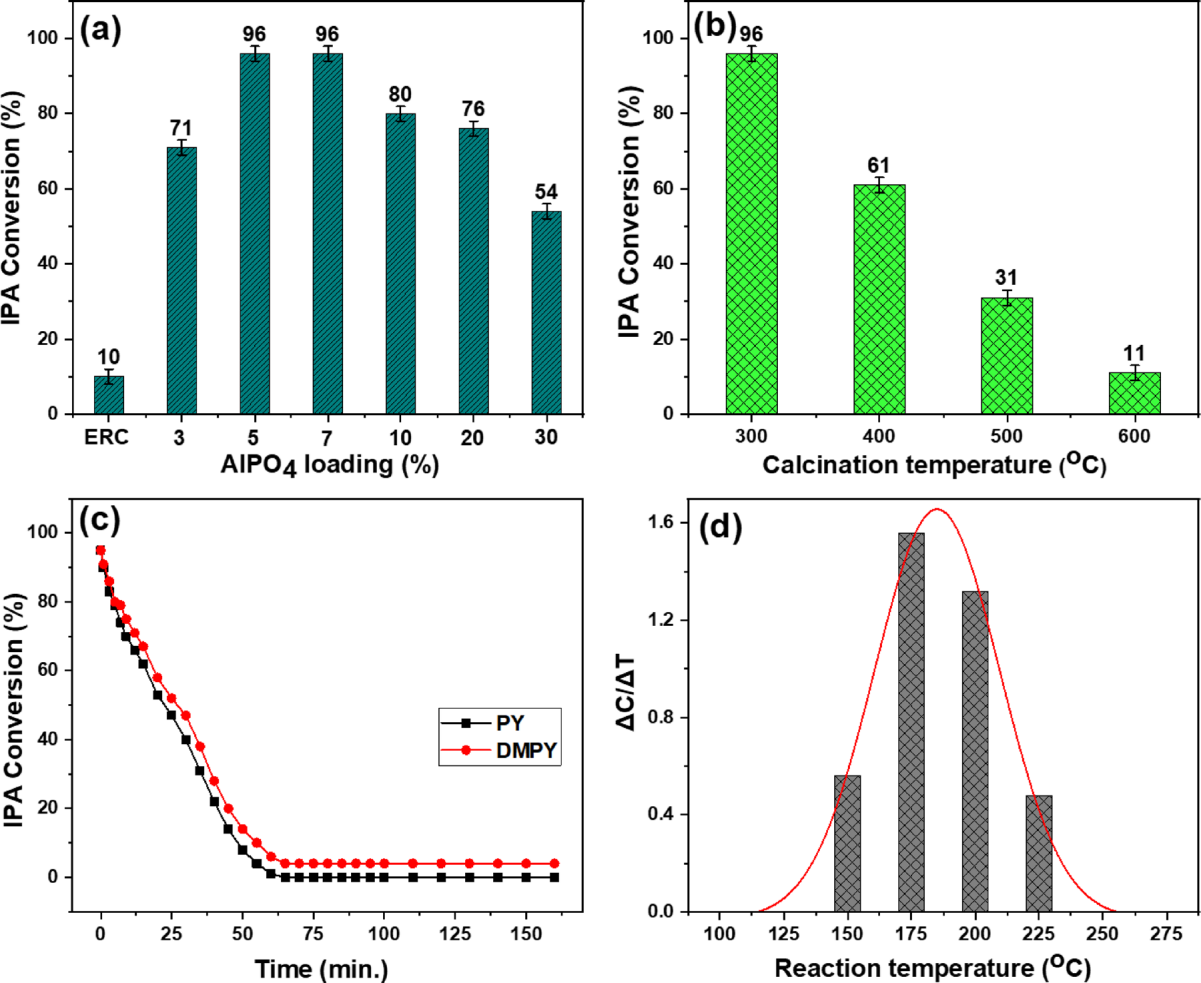
IPA dehydration over **(a)** untreated ERC and 3–30% AlPO_4_/ERC nanocomposites calcined at 300 °C, and **(b)** the 5% AlPO_4_/ERC nanocomposite calcined at different temperatures; **(c)** relationship between the IPA conversion and exposure time to PY and DMPY over the 5% AlPO_4_/ERC nanocomposite at a reaction temperature of 150 °C; and **(d)** PY desorption profile as a function of reaction temperature for the pre-saturated 5% AlPO_4_/ERC nanocomposite.

Previous studies have shown that methanol dehydration to DME is strongly influenced by the acidity of solid nanocomposites, particularly the type, strength, and distribution of acid sites^12,21^. The surface acidity of the produced nanocomposites was evaluated using IPA dehydration as a probe reaction at 150 °C. The results are shown in Fig. 6a. Chromatographic analysis confirmed propene as the sole product for all nanocomposites, indicating that the materials are predominantly acidic character^24,48^. The IPA conversion over untreated ERC was relatively low (10%) but increased significantly upon AlPO_4_ incorporation, reaching a maximum of 96% over the 5 and 7% AlPO_4_/ERC nanocomposites. However, a further increase in AlPO_4_ loading led to a gradual decline in conversion, which decreased to 54% at the highest loading (30 wt.%). These results suggest that moderate AlPO_4_ incorporation optimizes the density of accessible acid sites, with the 5 and 7% AlPO_4_/ERC nanocomposites exhibiting the highest concentrations of active acidic centers. Based on these results, the 5% AlPO_4_/ERC nanocomposite was selected for further study.

Figure 6b illustrates the effect of calcination temperature on the catalytic performance of the 5% AlPO_4_/ERC nanocomposite during IPA dehydration. IPA conversion decreases markedly with increasing calcination temperature, reaching only 11% for the nanocomposite calcined at 600 °C. This deactivation is attributed to reorganization and thermal degradation of surface phosphate species, which results in a decrease in surface area and a loss of active acid sites. Consequently, both Brønsted and Lewis acidity decrease because of partial decomposition and migration of phosphate groups, resulting in a significant reduction in total surface acidity^32,42^.

Although IPA dehydration provides a measure of total acidity, it does not distinguish between the types and strengths of the acid sites^45^. Therefore, additional chemisorption studies using PY and DMPY were performed at 150 °C to probe the nature of the acid sites on the 5% AlPO_4_/ERC nanocomposite (Fig. 6c). The gradual decrease in IPA conversion with increasing exposure time to PY and DMPY indicates progressive poisoning of the acid sites, eventually reaching a steady state corresponding to complete site saturation. The relatively small difference between the PY and DMPY profiles suggests that Lewis’s acid sites are less abundant than Brønsted acid sites.

Additionally, the distribution of acid-site strengths was evaluated by pre-adsorbing pyridine onto the most active nanocomposite (5% AlPO_4_/ERC) in an evacuated desiccator for 5 days. As shown in Fig. 6d, IPA dehydration was subsequently tested at increasing temperatures to monitor PY desorption behavior. The results indicate that most pyridine species desorb between 150 and 225 °C, confirming that the nanocomposite surface predominantly contains acid sites of intermediate strength.

### Catalytic dehydration of methanol to DME

**Fig. 7:**
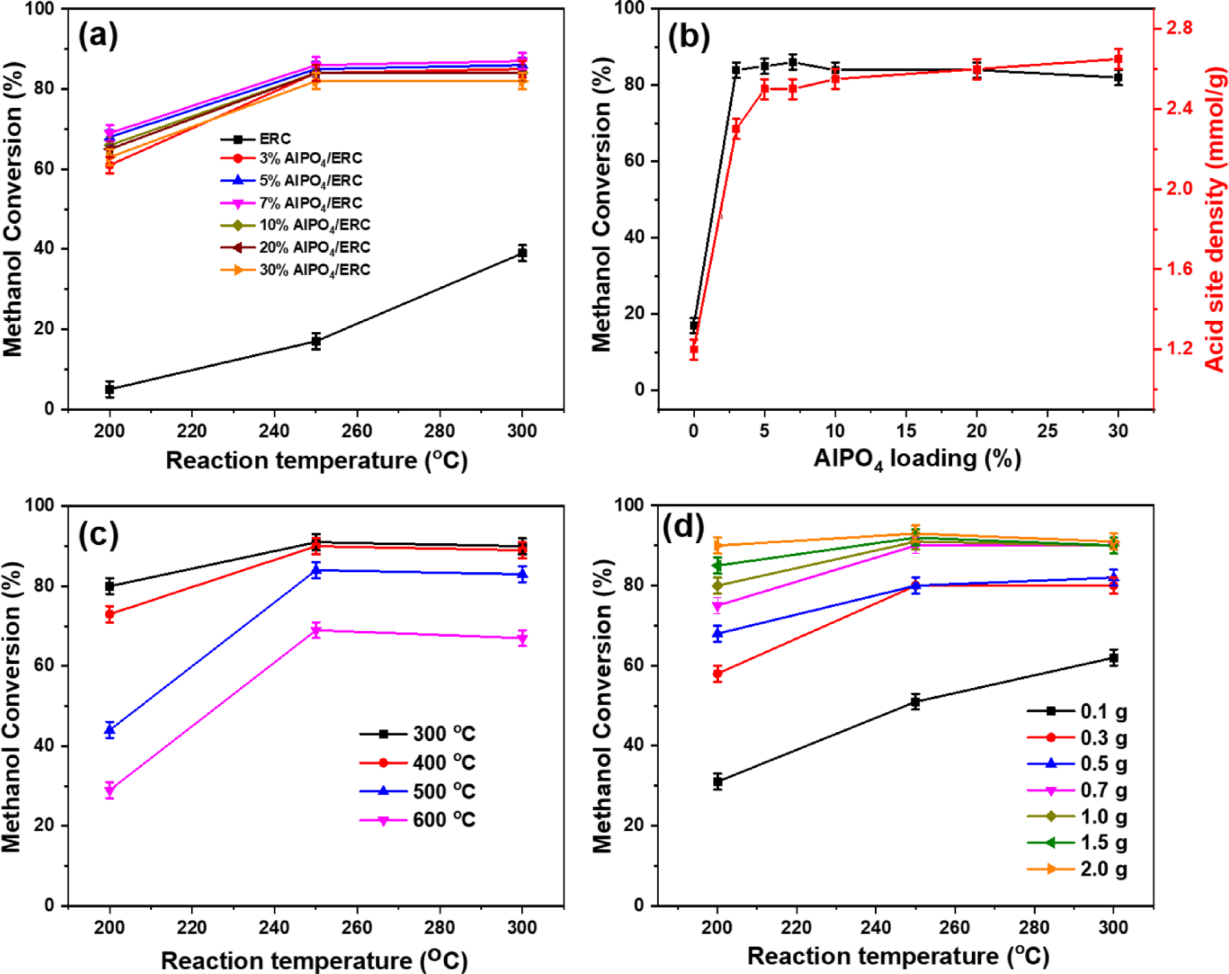
Methanol conversion as a function of reaction temperature over **(a)** untreated ERC and 3–30% AlPO_4_/ERC nanocomposites calcined at 300 °C; **(b)** relationship between the catalytic activity and the acid-site density as a function of AlPO_4_ loadings; **(c)** the 5% AlPO_4_/ERC nanocomposite calcined at different temperatures; and **(d)** the effect of catalyst mass on the performance of 5% AlPO_4_/ERC during methanol dehydration.

Vapor-phase dehydration of methanol to DME was investigated over pristine ERC and AlPO_4_-modified ERC nanocomposites (3–30% wt.%) calcined at 300 °C, at reaction temperatures of 200–300 °C. As shown in Fig. 7a, all catalysts exhibited 100% selectivity to DME with no detectable by-products, indicating that AlPO_4_ modification does not promote side reactions but instead enhances selective dehydration pathways. Pristine ERC showed limited activity, with methanol conversion increasing only from 5% at 200 °C to 39% at 300 °C, whereas AlPO_4_ incorporation led to a substantial improvement in catalytic efficiency. The 5% and 7% AlPO_4_/ERC catalysts achieved the highest conversions, 85% and 86% at 250 °C, respectively. This pronounced enhancement demonstrates that AlPO_4_-induced acidity modulation primarily increases the density of accessible weak and intermediate acid sites, which are the most active centers for selective methanol dehydration. In addition, the absence of by-products, together with the stable conversion trends, indicates that the modified catalysts suppress undesired secondary reactions and maintain high selectivity under the reaction conditions. The similar performances of the 5 and 7% AlPO_4_/ERC nanocomposites further confirm that an optimal acid-site distribution, rather than total acidity, governs catalytic efficiency. These results collectively demonstrate that AlPO_4_ modification provides a more effective and selective catalytic surface than unmodified ERC. The Fe_2_O_3_ phases present in the pristine ERC may contribute weak acidic and redox properties; however, the methanol dehydration reaction is primarily governed by the surface acid sites generated upon AlPO_4_ incorporation. The significant enhancement in catalytic performance observed for the AlPO_4_/ERC catalysts suggests that the increased acidity resulting from AlPO_4_ loading plays a more dominant role in DME production than the iron oxide species. Figure 7b illustrates the effect of AlPO_4_ loading on the acid-site density and catalytic performance of the AlPO_4_/ERC catalysts during methanol dehydration to DME at a reaction temperature of 250 °C. Pure ERC exhibited the lowest acid-site density, approximately 1.2 mmol/g, indicating the weak intrinsic acidity of the natural clay material. After AlPO_4_ loading, the acid-site density increased sharply to approximately 2.3–2.5 mmol/g at low loading percentages, confirming the successful generation of additional active acid sites on the clay surface. This enhancement in acidity is mainly attributed to the formation of Lewis and Brønsted acidic centers associated with phosphate species and to the strong interactions between AlPO_4_ and the ERC support. Further increases in the AlPO_4_ loading percentage resulted in only a slight increase in acidity, reaching 2.55–2.65 mmol/g at higher loadings and indicating the formation of an acidity plateau because of surface saturation and partial agglomeration of AlPO_4_ particles. Figure 7b also demonstrates a strong correlation between acid-site density and methanol conversion during the dehydration reaction. The sharp increase in acidity after AlPO_4_ loading was accompanied by a significant increase in methanol conversion, indicating that the dehydration of methanol to DME strongly depends on the availability of active acid sites. These acidic centers facilitate the adsorption and activation of methanol molecules, thereby accelerating the dehydration process and improving catalytic activity. At the low acidity, as observed for pure ERC, the limited number of active sites restricts methanol adsorption and results in lower catalytic performance. In contrast, the AlPO_4_-loaded catalysts exhibited substantially higher acidity and consequently, higher methanol conversion because of the increased number of accessible active sites. However, although the acid-site density continued to increase slightly at higher AlPO_4_ loading percentages, the improvement in methanol conversion became less pronounced. This behavior suggests that once a sufficient number of acidic sites has been generated, catalytic activity approaches an optimal value and further increases in acidity contribute only marginally to the reaction performance. The enhanced catalytic performance is therefore attributed to the higher density of accessible acid sites, particularly weak and intermediate-strength acid sites, which are known to be highly active for methanol dehydration^22,46^. The limited enhancement at higher loading percentages may result from partial pore blockage, reduced surface accessibility, or aggregation of AlPO_4_ particles, which can decrease the effectiveness of newly formed acid sites. Therefore, the results indicate that moderate AlPO_4_ loading levels provide an optimal balance between acidity and surface accessibility, making these catalysts highly effective for methanol dehydration to DME. Based on these findings, the 5% AlPO_4_/ERC nanocomposite was selected for further study.

As shown in Fig. 7c, the effect of calcination temperature on the catalytic performance of the 5% AlPO_4_/ERC nanocomposite was further assessed over the same reaction-temperature range (200–300 °C). At all reaction temperatures, the nanocomposite that was calcined at 300 °C exhibited the highest catalytic activity. In contrast, a further increase in calcination temperatures beyond 300 °C resulted in a marked decrease in methanol conversion. This deactivation is primarily attributed to the loss of accessible acid sites and the decrease in specific surface area caused by sintering, structural rearrangement, or partial collapse of phosphate species at high temperatures.

The 5% AlPO_4_/ERC nanocomposite was used to examine the effect of nanocomposite mass on methanol conversion and DME selectivity over the temperature range of 200–300 °C, as shown in Fig. 7d. At 200 °C, methanol conversion and DME production increased significantly from 31 to 90% as the nanocomposite mass increased from 0.1 to 2.0 g. This enhancement is attributed to the increased number of accessible active acid sites at higher nanocomposite loadings, which improves the interaction between methanol molecules and the nanocomposite surface. Methanol conversion remained nearly constant at the higher temperatures of 250 and 300 °C, at 93% and 91%, respectively, suggesting that the reaction was close to equilibrium under these conditions. Based on these findings, a nanocomposite mass of 2.0 g and a reaction temperature of 200 °C were selected as the optimal conditions for further study.

**Fig. 8:**
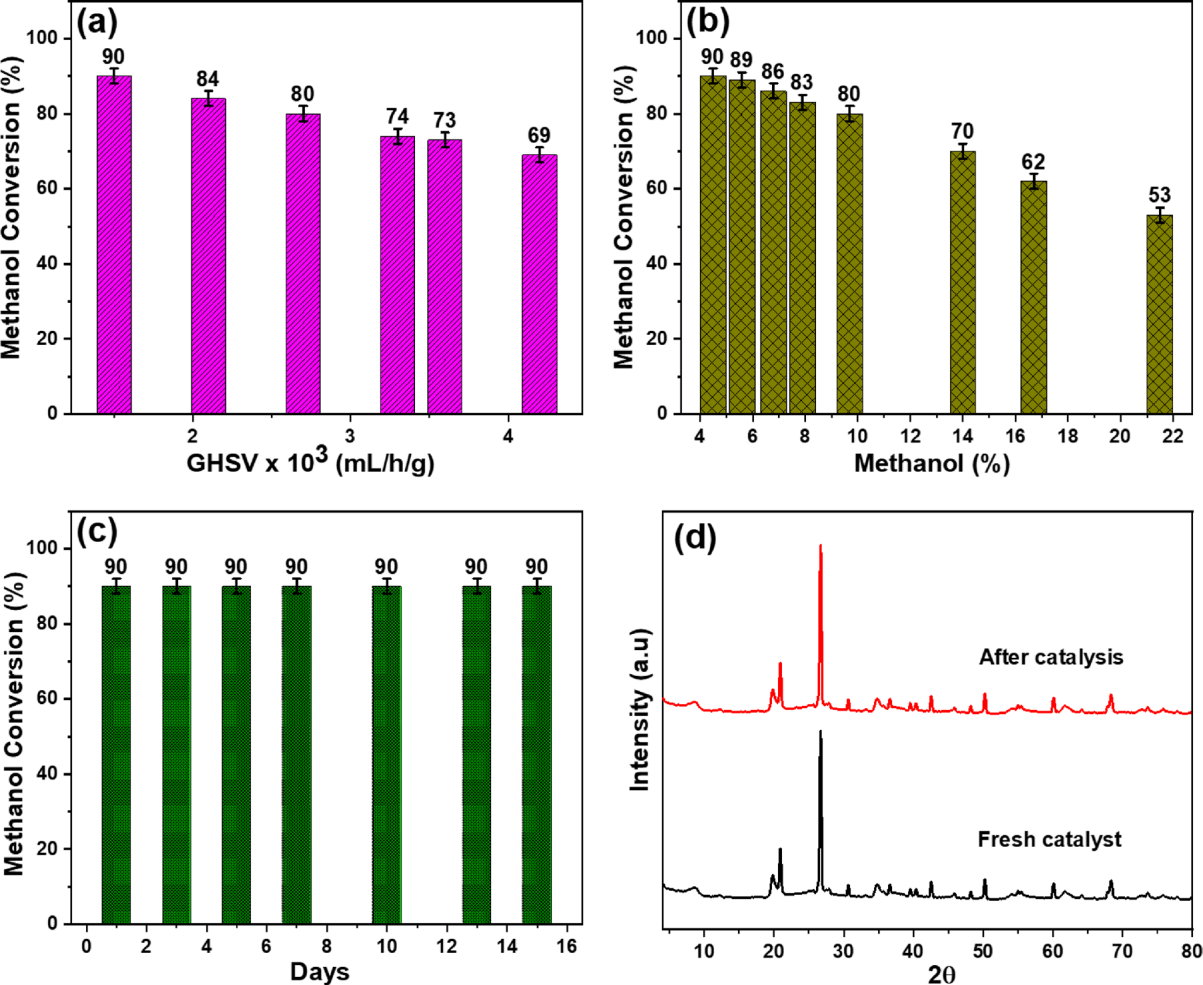
**(a)** Influence of gas hourly space velocity (GHSV), **(b)** effect of methanol feed concentration, **(c)** long-term stability of the 5% AlPO_4_/ERC nanocomposite during methanol dehydration, and **(d)** XRD patterns of the fresh and spent 5% AlPO_4_/ERC nanocomposite.

At 200 °C, the effect of gas hourly space velocity (GHSV) on the catalytic activity of the most active nanocomposite (5% AlPO_4_/ERC) was assessed, and the results are shown in Fig. 8a. Methanol conversion decreased significantly from 90 to 69% as the GHSV was increased from 1500 to 4200 mL h^−1^ g^−1^, whereas DME selectivity remained constant at 100% under all examined conditions. This decrease in conversion is attributed to the shorter residence time of the reactant molecules on the nanocomposite surface at higher space velocities, which limits their interaction with the active acid sites^47^.

As shown in Fig. 8b, the effect of methanol concentration in the feed stream on catalytic performance was examined at 200 °C. Methanol conversion gradually decreased to 53% as the methanol concentration increased from 4.5 to 21.5%; however, DME selectivity remained at 100%. This trend is attributed to the competitive adsorption of methanol molecules on a limited number of active acid sites, leading to surface saturation and a decrease in the number of accessible reactive sites^12^.

Catalyst stability is a crucial factor when assessing the practical application of methanol dehydration to DME. Accordingly, the long-term stability of the 5% AlPO_4_/ERC nanocomposite was evaluated over 15 days on stream at 200 °C. Throughout the test, the nanocomposite maintained a consistent methanol conversion of approximately 90% with 100% selectivity to DME, as shown in Fig. 8c. This result indicates outstanding resistance to deactivation under the conditions under investigated conditions. The structural stability of the nanocomposite was further validated by post-reaction characterization. The XRD patterns of the fresh and spent nanocomposites (Fig. 8d) show no discernible changes in their diffraction characteristics, indicating that the crystalline structure of the nanocomposite is unaffected by prolonged use. Furthermore, the FTIR spectra of the fresh and spent 5% AlPO_4_/ERC nanocomposite after methanol dehydration to DME (Figure S1) indicate that the catalyst structure remained largely unchanged during the reaction. The broadband at 3600–3200 cm^−1^ became more intense after catalysis, suggesting increased adsorption of the water produced during methanol dehydration. The bands appearing at 2360 and 2360 cm^−1^ are typically assigned to the asymmetric stretching vibration of atmospheric CO₂ adsorbed on the catalyst surface or present in the optical path of the FTIR instrument. The enhanced band near 1630 cm⁻^1^ further confirms the presence of adsorbed water. In the fingerprint region, the characteristic band at 1000–1100 cm^−1^, assigned to Si–O–Si, Si–O–Al, P–O, and Al–O–P vibrations, remained essentially unchanged, indicating preservation of the AlPO_4_/ERC framework. Collectively, these findings confirm the exceptional catalytic stability and durability of the 5% AlPO_4_/ERC nanocomposite during methanol dehydration to DME.

Finally, as shown in Table 3, the catalytic performance of the 5% AlPO_4_/ERC nanocomposite developed in this work was compared with that of previously reported nanocomposites. The comparison highlights several key advantages of the present system, including a relatively low operating temperature, high methanol conversion (90%), complete selectivity toward dimethyl ether (100%), and excellent long-term stability. The sustainability, scalability, and practical applicability of the proposed nanocomposite system are further enhanced by the use of ERC, a naturally occurring and inexpensive material that is readily available in large quantities.

**Table 3:** Comparison of the catalytic performances of the 5% AlPO_4_/ERC nanocomposite with those reported in the literature for the dehydration of methanol to DME.

Nanocomposite	Reaction temperature (ºC)	Methanol conversion (%)	DME selectivity (%)	Reference
AP1.5	300	81.9	99.9	^48^
5% P/γAl_2_O_3_	240	42.0	75.0	^19^
AlPO_4_/ ZSM-5	300	84.0	82.5	^22^
SAPO-46	350	80.0	93.0	^49^
Silica-titania and modified γ-Al_2_O_3_ with phosphorus	300	83.5	89.8	^50^
10% water vapor/AlPO_4_	300	82.0	99.0	^51^
1% H_2_SO_4_/0.1Zr AlPO_4_/TRI	250	89.0	100	^47^
7% Al_2_O_3_/ACP	300	85.0	100	^46^
5% AlPO_4_/ERC	200	90.0	100	This work

### Reaction mechanism of methanol dehydration

**Scheme 2:**
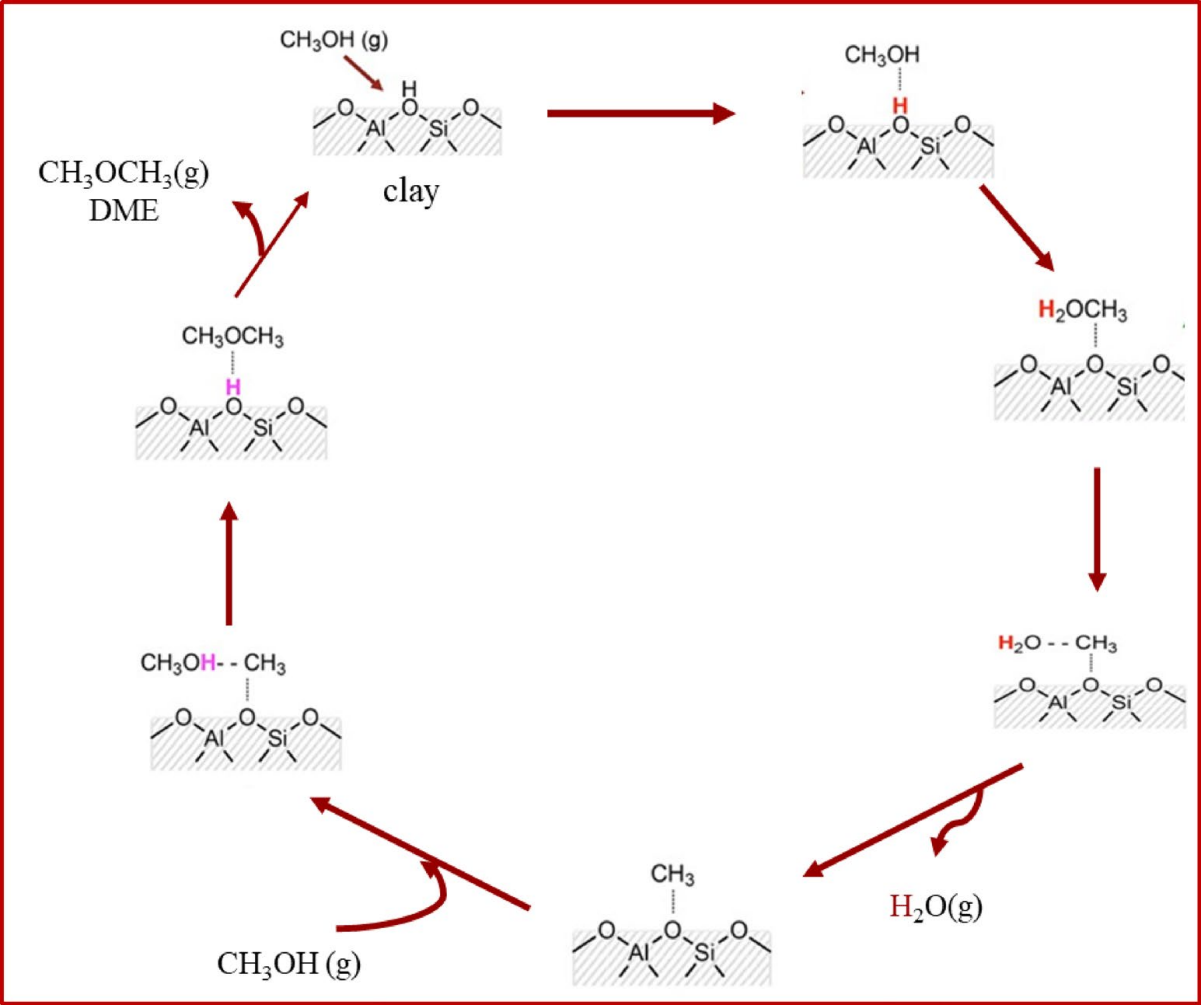
Proposed reaction mechanism for the dehydration of methanol to DME over a clay nanocomposite.

The catalytic dehydration of methanol to DME on an acidic surface, such as a clay nanocomposite (represented by the Al–O–Si framework in Scheme 2, proceeds primarily through a dissociative mechanism. Gaseous methanol (CH₃OH(g)) first adsorbs onto a Lewis or Brønsted acid site, typically associated with an aluminum atom, forming an adsorbed methanol species (top right of the diagram). In the dissociative pathway, this adsorbed methanol loses a water molecule, generating a surface-bound methoxy group (–OCH₃) attached to the aluminum site, as shown in the lower right pathway leading to the CH₃–O–Al species. A second methanol molecule then performs a nucleophilic attack on the carbon atom of this surface methoxy intermediate^52^. The oxygen atom of the incoming methanol bonds to the methyl group while the surface methoxy species departs, directly yielding DME (CH₃OCH₃), regenerating the acid site, and releasing water as a byproduct. The scheme also illustrates alternative or parallel associative routes (left side), in which two methanol molecules interact in a more concerted manner on the surface, forming protonated intermediates such as CH₃OH₂⁺–CH₃ or hydrogen-bonded complexes, before eliminating water and producing DME. The red arrows highlight the cyclic nature of the catalytic process, in which the surface sites (shaded Al–O–Si clusters) are continuously regenerated, allowing sustained turnover. This dissociative route is particularly favored on clay nanocomposites because the stable surface methoxy intermediate lowers the activation energy for C–O bond formation while facilitating water desorption. Overall, the process converts two CH₃OH molecules into CH₃OCH₃ and H₂O, with the surface acidity playing a critical role in both adsorption and the key nucleophilic step.

## Conclusions

AlPO_4_ modification effectively tailors the acidity and porosity of Egyptian red clay (ERC) for methanol dehydration to DME without altering its aluminosilicate framework. The 5% AlPO_4_/ERC catalyst exhibits the best performance, achieving 90% methanol conversion with 100% selectivity, because of an optimal balance of weak and intermediate acid sites (~ 2.5 mmol g^−1^) and high surface accessibility. Higher AlPO_4_ loadings decrease activity because of pore blockage and reduced site accessibility, despite their slightly higher acidity. The reaction proceeds through acid-site-mediated methanol activation via surface methoxy intermediates, indicating that catalytic performance is governed by the nature and accessibility of the acid site rather than by iron species. Long-term testing over 15 days confirms excellent stability without deactivation. Overall, the results demonstrate that optimizing the acid-site distribution is more important than maximizing total acidity, establishing a clear structure-acidity-performance relationship for efficient DME production.

## Supplementary Information

Below is the link to the electronic supplementary material.

Supplementary Material 1

## Data Availability

All data generated or analysed during this study are included in this published article (and its Supplementary Information files).
