# Supplementary material for: Aluminum phosphate-modified red clay as a stable nanocomposite catalyst for the selective conversion of methanol to dimethyl ether
Source: Sci Rep. 2026 Aug 4;16:24009. doi: 10.1038/s41598-026-63442-x (PMC13439434; doi:10.1038/s41598-026-63442-x)
Supplement: Supplementary file 1 [file 41598_2026_63442_MOESM1_ESM.docx]

**Aluminum phosphate–modified red clay: a sustainable, low-cost, and highly stable nanocomposite for the efficient and selective conversion of methanol to dimethyl ether Mohamed N. Goda^1,2^, Abd El-Aziz A. Said^1^*, Asmaa Mohamed^1^, Esraa Magdy^1^, Mohamed Abd El-Aal^1^**

**^1^Catalysis and Surface Chemistry Laboratory, Chemistry Department, Faculty of Science, Assiut University, Assiut, 71516, Egypt**

**^2^Department of Chemistry, College of Science, Imam Mohammad Ibn Saud Islamic University (IMSIU), Riyadh, 11623, Saudi Arabia**

***Corresponding author Email:** [**a.a.said@aun.edu.eg**](mailto:a.a.said@aun.edu.eg)**;** [**aasaid55@yahoo.com**](mailto:aasaid55@yahoo.com) **(Abd El-Aziz A. Said)**

**Table S1**. Chemical analysis of the red clay samples (wt. %).

| **Compounds** | **SiO_2_** | **Al_2_O_3_** | **Fe_2_O_3_** | **TiO_2_** | **CaO** | **K_2_O** | **MgO** | **MnO_2_** | **Na_2_O** | **P_2_O_5_** |
| --- | --- | --- | --- | --- | --- | --- | --- | --- | --- | --- |
| **% wt./wt.** | 34.83 | 31.51 | 9.85 | 1.70 | 1.21 | 1.17 | 1.11 | 0.89 | 0.12 | 0.08 |

**Figure S1**. FTIR spectra of the fresh and spent 5 wt.% AlPO_4_/ERC nanocomposite.
